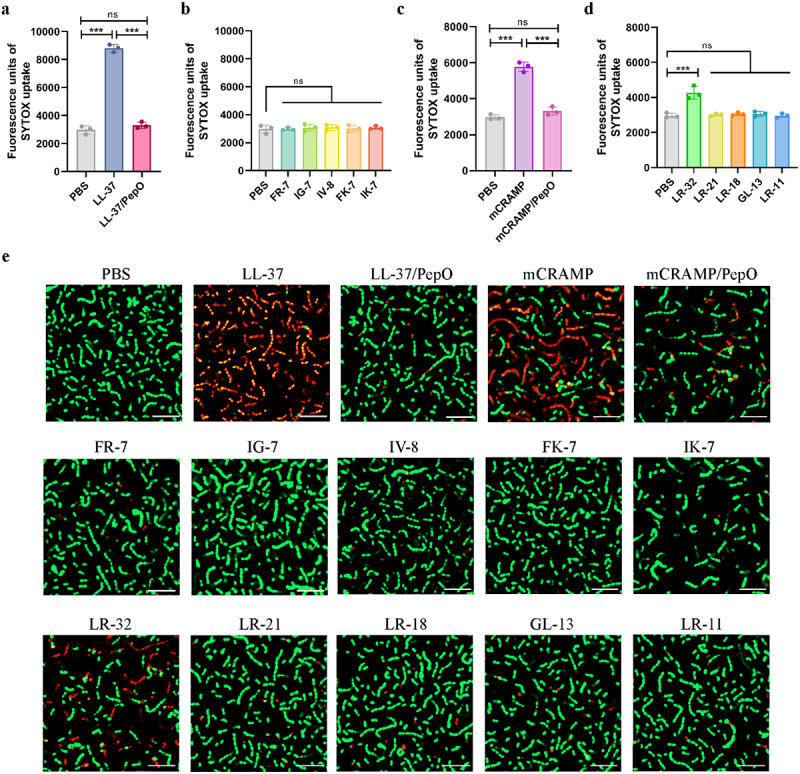# Correction

**DOI:** 10.1080/21505594.2023.2289756

**Published:** 2023-12-13

**Article title**: Endopeptidase O promotes Streptococcus suis immune evasion by cleaving the host- defence peptide cathelicidins

**Author name**: Mingjie Jin, Siyu Liang, Jing Wang, Huihui Zhang, Yueling Zhang, Wanjiang Zhang, Siguo Liu, and Fang Xie

**Journal**: *Virulence*

**DOI**: https://doi.org/10.1080/21505594.2023.2283896

When the article was originally published the figure 4 was published with minor error in the figure parts, while the process of combining images of Figure 4e, an image of IG-7 taken by CLSM was incorrectly introduced as the image of IK-7 due to negligence. This change has now been rectified with the correct figures (as mentioned below), and the corrected article has been republished now.

**Figure:**